# Appendicitis and Pseudo-Appendicitis in Certain Infectious Diseases.—I

**Published:** 1911-06-03

**Authors:** 

**Affiliations:** Physician to the Hospital "Des Enfants Malades," Professor in the Faculty of Médecine, Member of the Academy of Médecine, Paris.


### Hospital Clinics.

### APPENDICITIS AND PSEUDO-APPENDICITIS IN CERTAIN INFECTIOUS DISEASES.—I.

A Lecture by Dr. HUTINEL, Physician to the Hospital " Des Enfants Malades," Professor in the Faculty of Médecine, Member of t he Academy of Médecine, Paris.

When one examines methodically the abdomen of patients suffering from infectious diseases it sometimes happens that one finds a certain amount of sensitiveness, or even acute pain, in the right iliac fossa at McBurney's point. Is one in presence of an attack of appendicitis, or is it rather only pseudo-appendicitis, and in this case what is the prognosis and what treatment should be applied? Such are the questions which I propose to study with you to-day. However, do not expect me to pass in review all the various infectious states which, in children, may react on the appendix. I will simply describe a few typical cases which I have had occasion to observe and some of which caused me no little embarrassment. The appendix, as you are aware, is lined with lymphoid tissue similar to that which, in the throat, forms the tonsils and adenoid vegetations (Waldeyer's lymphatic circle), and to that which constitutes the intestinal follicles and Peyer's patches.

It is, therefore, quite possible that any infection which affects the lymphoid tissue may also attack the appendix. On the other hand, the organ is simply a diverticle of the intestine and we can easily understand that any disease of the latter may affect it also. Its circulation may be modified by various causes; the glands of its peritoneal fold may become infected and contribute in producing the appendicular reaction we are studying. In little girls the neighbourhood of the genital organs explains the propagation of uterine and tubo-ovarian lesions to the appendix. Lastly, peritoneal infection, as, for example, in certain forms of tuberculous peritonitis, may be localised in the right iliac fossa. I shall, however, do no more than simply mention this point, for I intend to speak of tubercular appendicitis and pseudo-appendicitis in a subsequent lecture.

### Measles and Appendicitis.

We have, at this moment, in our measles ward a little girl who was taken suddenly ill with vomiting and fever. On examination I found pain and diffuse swelling in the whole right iliac fossa. The next day the rubeolic rash appeared and two days later all the signs of appendicitis had subsided. Now, what is going to happen in this case? I am not in a position to say for certain, but I believe that all has become normal again. Eight days ago another little girl complained of sudden pain at McBurney's point; there was diffuse swelling of the whole right fossa with tympanitis, vomiting supervened. The next day the typical rash of measles appeared and the symptoms, which the day before had caused me much anxiety, became less marked and at the present moment have disappeared altogether.

The following is a much more serious caes. About a year ago I saw a South American girl of fifteen who, on several occasions, had suffered from attacks of pain in the right iliac fossa. During the four days previously she had had a temperature hovering round 102° F. when she was taken suddenly with vomiting and abdominal pain, the latter soon localising at McBurney's point. The right fossa was swollen and dull on percussion; the abdomen was distended, and the face pinched. The family doctor in presence of a temperature running up to 104° F. and a small and rapid pulse, asked for a consultation and a hospital physician was called who, after a careful examination of the patient, advised an immediate operation. Before the latter could be arranged for, however, small red spots appeared on the face, the neck, the soft palate, and soon spread to the body and limbs. I was called the next day and found that I had to do with a typical case of measles. Although the local symptoms had become rather worse, I reassured the parents, for I knew that, when the rash has completely come out, the local symptoms generally disappear in a short period of time. As a matter of fact that same evening the temperature fell to 101°, the general state improved, and everything seemed to announce an early recovery. A few days later, however, the temperature went up again; pus had collected in the right fossa and had to be evacuated. The patient recovered rapidly.

### An Attempt at an Explanation.

Now let us see what took place in the cases I have just described. My first thought was that, in these patients, the appendix had been already damaged by some anterior cause and that, under the influence of the attack of measles, it had become the seat of more or less intense congestion. You are aware, of course, that the existence of some old lesion in any part of the body is very apt to become congested under the influence of any new infection; that if, for instance, an individual having some scar or cicatrix, contracts scarlet fever, measles, varicella, or syphilis, the eruptive elements, maculæ, bullæ, roseola, etc., present their maximum of intensity in the region of the scar, that is, in the region where the circulation has undergone modification. You are also aware that the morbillous rash takes place not only on the skin, but on the peritoneum also, and that one of the first symptoms of the disease consists in a peculiar peritoneal friction sound. We can very well admit, therefore, that measles gives rise, in patients whose appendix has already been damaged, to marked local congestion. But, on the other hand, it is also quite possible that it opens the door to certain secondary infections affecting especially the intestine. I have seen most serious symptoms, terminating fatally, in a child who contracted measles during an attack of colitis, and I have no doubt that the symptoms were due to an increase in virulence of the intestinal germs under the influence of the general infection.

When, therefore, at the outset of measles you find in your little patients the presence of appendicular symptoms, do not be in a hurry to advise a surgical intervention; forbid all food, apply ice to the abdomen, wrap their limbs in cottonwool, and await events. In the majority of cases the local reaction will diminish as soon as the rash has thoroughly come out, and even if an abscess should form try and put off an operation until the height of the disease has passed.

In scarlet fever appendicular symptoms are not at all exceptional. One of the assistant physicians of the hospital, Dr. Kauffmann, has just written a very interesting work on this subject. He connects with the appendix certain cases of early vomiting and most of the cases of acute peritonitis coming on in the course of the disease. Perhaps even, in his opinion, some of the cases of malignant scarlatina are due to septic appendicular peritonitis. He says one may observe, when the rash appears, that the right iliac fossa often presents a certain sensitiveness and sometimes actual pain on pressure. It is therefore probable that the appendix is affected much more frequently in scarlet fever than one may imagine, and although the symptoms are generally of a mild character, they may perhaps pass into a chronic state, thus favouring the development of acute appendicitis at a later date. In some cases the lesions give rise to perforation from the very onset. Dr. Kauffmann describes these lesions as a tumefaction of the follicles and glands in the appendicular peritoneum, and in microscopical ulcerations which are, as a rule, very marked. These facts should encourage careful study of appendicular symptoms in scarlet fever. Dr. Jalaguier tells me that he attended a child who developed, during the course of scarlet fever, an extremely serious attack of appendicitis, which however got well under simple medical treatment, the appendix being removed five weeks later. Dr. Veau has also published three similar cases. You need not, therefore, be very much surprised when you meet symptoms of appendicitis in the course of scarlet fever; you must suppress all food, apply ice to the abdomen, and order the strictest immobility to the patient; a surgical intervention being only indicated in case of extreme urgency.*

Some authorities consider that influenza plays an important rôle in the genesis of appendicitis. It is quite certain that the onset of the latter may present symptoms identical with those of influenza; I have had several times the occasion to observe such cases. A few months ago I was called to see a girl of seventeen who, after an ordinary attack of influenza, had suffered for several days from abdominal pain; the intestinal form of influenza was the diagnosis I was told. Be always on your guard and mistrust such a diagnosis; it is nearly always erroneous. As soon as I saw the patient I was struck by the alteration of the facies; the nose was pinched, the eyes hollow, the whole face presented an anxious expression; the temperature hovered round 103° F., the pulse 140, the abdomen was distended and painful all over, but the maximum of pain was situated at McBurney's point. By digital examination of the rectum, which I strongly advise you always to make in such cases, I felt the peritoneum bulging in the pelvic cavity. It was a case of fulminating pelvic appendicitis. A surgeon was called immediately, but he found the general state so bad that he dared not operate and the patient died that same evening. Was it a localisation of Pfeiffer's bacillus in the appendix, or was it an old appendicular infection revived by influenza? I am not able to say; but happily such cases are quite exceptional and in the majority of cases appendicular symptoms in influenza remain quite mild.

Vaccination may also, as Russel has shown, give rise to appendicular reaction. Jalaguier has related a case of appendicitis coming on in the course of varicella and ending favourably. Little girls suffering from mumps sometimes present a certain fulness and pain in the right iliac fossa; these symptoms are due to ovaritis and when they appear after the parotid tumefaction the diagnosis is easy, but when they precede it, as in a case mentioned by Dr. Mery, the diagnosis of appendicitis is almost invariably made.

The connection between typhoid fever and appendicitis is much more interesting and I shall dwell rather longer on this part of the subject. Appendicitis may supervene at any period of the disease; I will, however, do no more than just mention the peritonitis of appendicular origin which may appear as a complication in the course of typhoid and will confine myself to peritonitis coming on at its very onset. I was very much struck by the following case. I was called one day to see a pretty little girl of fourteen, habitually in very good health, who a few days previously had suddenly been taken with vomiting and abdominal pain; there was diffuse swelling in the right fossa, constipation, and fever. The diagnosis of appendicitis seemed quite clear and I applied the classic treatment, informing the parents that it would be advisable a little later on to remove the appendix in order to prevent the return of any similar attacks. The next day the temperature still remained high but the pain had greatly diminished, although the abdomen was still distended. A few days later rose spots made their appearance and a typical attack of enteric ensued which ran a quite usual and normal course. It is needless to state that nothing more was said about an operation.

*(To be continued.)*

